# Impact of RSV Infection in Transplant and Immunocompromised Population: Incidence and Co-Infections: Retrospective Analysis of a Single Centre

**DOI:** 10.3390/jcm14134803

**Published:** 2025-07-07

**Authors:** Paolo Solidoro, Antonio Curtoni, Sara Minuto, Nour Shbaklo, Francesco Giuseppe De Rosa, Alessandro Bondi, Francesca Sidoti, Filippo Patrucco, Elisa Zanotto, Silvia Corcione, Massimo Boffini, Matteo Marro, Cristina Costa, Rocco Francesco Rinaldo

**Affiliations:** 1 Division of Respiratory Medicine, Cardiovascular and Thoracic Department, AOU Città della Salute e della Scienza di Torino, University of Turin, 10126 Torino, Italy; psolidoro@cittadellasalute.to.it (P.S.); roccofrancesco.rinaldo@unito.it (R.F.R.); 2 Medical Sciences Department, University of Turin, 10126 Torino, Italy; nour.shbaklo@unito.it; 3 Division of Virology, Department of Public Health and Pediatrics, AOU Città della Salute e della Scienza di Torino, University of Turin, 10126 Torino, Italy; antonio.curtoni@gmail.com (A.C.); alessandro.bondi@unito.it (A.B.); francesca.sidoti@unito.it (F.S.); elisa.zanotto@unito.it (E.Z.); cristina.costa@unito.it (C.C.); 4 Unit of Infectious Diseases, Department of Medical Sciences, University of Turin, 10149 Torino, Italy; francescogiuseppe.derosa@unito.it (F.G.D.R.); silvia.corcione@unito.it (S.C.); 5 Respiratory Diseases Unit, Medical Department, AOU Maggiore della Carità di Novara, 28100 Novara, Italy; 6 Division of Geographic Medicine, Tufts University School of Medicine, Tufts University, Boston, MA 02111, USA; 7 Cardiac Surgery Division, Surgical Sciences Department, AOU Città della Salute e della Scienza di Torino, University of Turin, 10126 Torino, Italy; massimo.boffini@unito.it (M.B.);

**Keywords:** respiratory syncytial virus, lung transplantation, viral respiratory infections, vaccine, bronchoalveolar lavage

## Abstract

Respiratory syncytial virus (RSV) represents one of the main respiratory infections found among immunocompromised patients. **Objective**: The study analyzes the incidence of RSV infection in different populations of immunocompromised patients as organ transplant recipients (lung, other solid organs, hematopoietic stem cells) and oncologic patients (solid organ malignancy and hematological malignancy) compared to a group of non-immunocompromised patients. We also assessed the prevalence of viral, bacterial, and mycotic coinfection. Moreover, we aimed at evaluating the efficacy of ribavirin treatment in terms of mortality reduction. **Methods**: We conducted a retrospective analysis on a total of 466 transplant patients undergoing bronchoscopy with bronchoalveolar lavage for suspected viral disease or surveillance between 2016 and 2023, compared to 460 controls. **Results**: The incidence of RSV was significantly higher in immunocompromised patients, particularly in those with lung and bone marrow transplants. Among RSV+ patients, a higher prevalence of viral (influenza virus), bacterial (*S. pneumoniae*, *M. pneumoniae*, *Nocardia* spp.), and fungal (*Aspergillus* spp.) coinfections were observed. The efficacy of ribavirin in reducing mortality did not show significant differences compared to supportive therapy alone. **Conclusions**: The results of our exploratory study suggest that immunocompromised patients are particularly vulnerable to RSV infection and coinfections. Our hypothesis-generating data warrant the need for future studies aimed at exploring preventive and therapeutic strategies for RSV infection in these high-risk patient groups.

## 1. Introduction

Lung transplantation represents the last therapeutic option for patients with various end-stage respiratory diseases [1]. One of the main challenges in transplant recipients’ management is airway infections. Among these, infections caused by non-influenza respiratory viruses, such as respiratory syncytial virus (RSV), are becoming increasingly significant [2]. Severe RSV infections have been associated with a decline in lung function (defined as a decrease in FEV1) and an increased risk of developing chronic lung allograft dysfunction (CLAD), which represents one of the main factors limiting the survival of lung transplant recipients [3,4,5,6].

RSV is one of the most commonly isolated community-acquired respiratory viruses, with incidence of up to 16% after lung transplant. The overall mortality for RSV among immunocompromised patients can be as high as 20%. Moreover, in lung transplant recipients, the overall mortality for RSV infections ranges from 10% to 20%. Acutely, RSV infection is associated with the development of bronchiolitis, lower respiratory infection, and respiratory failure [3,5].

Therapeutic strategies currently consist mainly of supportive care and steroids. Antiviral treatment options such as ribavirin, antibody-based treatments such as palivizumab, and intravenous immunoglobulins are of limited accessibility and have shown limited efficacy data and lack of evidence in lung transplant populations [7]. Ribavirin has shown in vitro activity against RSV, but although it is widely used in severe infection, its clinical benefit appears to be limited [3,8]. Given the obvious impact of RSV on society, in recent years, numerous studies aimed at developing a vaccine directed against this virus have been carried out. This has led, since mid-2023, to the approval by the European Medicines Agency (EMA) and the Agenzia Italiana del Farmaco (AIFA) of three types of vaccines. The available vaccines are based on the F glycoprotein of RSV in the stabilized prefusion conformation (PreF). There are two types: glycoprotein subunit vaccines (adjuvanted monovalent RSV vaccine [9] and PreF bivalent vaccine [10]) and mRNA vaccine [11].

The primary objective of this retrospective cohort study is to define the incidence of respiratory viral infections in specific immunocompromised patient populations (patients who have undergone lung, other solid organ, and bone marrow transplant, as well as oncology patients, divided into solid organ and hematological neoplasia for further comparison). Furthermore, the secondary exploratory objectives are to estimate the prevalence of bacterial, viral, and fungal co-infections with RSV and the difference in terms of mortality between patients with RSV treated with ribavirin versus patients treated with supportive therapy only.

## 2. Materials and Methods

### 2.1. Study Design

This retrospective observational study analyzed clinical and microbiological parameters of patients who underwent bronchoscopy with bronchoalveolar lavage (BAL) for the detection of respiratory syncytial virus (RSV) at AOU Città della Salute e della Scienza of Turin. The data were collected from January 2016 to December 2023. The data collected included demographic information, clinical details (such as immune status, whether the patient had undergone a transplant—lung, other solid organ, or hematopoietic stem cell), results from bacterial, viral, and fungal cultures on BAL, and galactomannan antigen levels. We retrieved this data from electronic medical records such as TrakCare (InterSystems Corp, Boston, MA, USA) and DNWeb (Dedalus S.p.a., Milan, Italy).

We identified a population of immunocompromised patients that included lung transplant recipients, other solid organ transplant recipients, hematopoietic stem cell transplant recipients, solid organ oncologic patients, and malignant hematologic patients. In addition, we included data from a control cohort of non-transplant and non-oncologic patients who underwent bronchoscopy due to suspicion of an infectious lung disease.

The decision of undertaking BAL was based on clinical judgment in the presence of worsening respiratory symptoms, new or progressive infiltrates based on chest radiography or computed tomography scans, and/or supporting biochemical and clinical data (e.g., fever). Moreover, the lung transplant patients also underwent surveillance BALs as per our center’s protocol (1, 4, 8, 12, 18, 24 months from transplant), independent of symptoms.

Two types of molecular panels were used to analyze the samples: GeneXpert Xpress^®^ Flu/RSV (Cepheid s.r.l., Milan, Italy) and Filmarray Respiratory Panel (BioMérieux S.p.A., Firenze, Italy). GeneXpert Cepheid Xpert Xpress^®^ Flu/RSV is a CE-IVD-certified test, with a specificity and sensitivity higher than 98% for RSV and influenza A and B. In most cases, the GeneXpert Cepheid was used as the first test, as it identifies the main pathogens capable of causing severe respiratory infections. In case of negativity, further analysis was carried out using Filmarray to extend the search. However, when rapid diagnosis was necessary for certain cases, Filmarray was used in the first place.

The identifiable pathogens from this panel include adenovirus, coronavirus 229E, HKU1, OC43 and NL63, Human Metapneumovirus, Human Rhinovirus/Enterovirus, influenza A, A/H1, A/H1-2009, A/H3, B, Parainfluenza 1–4, RSV, *Bordetella pertussis*, *Chlamydophila pneumoniae*, and *Mycoplasma pneumoniae*.

PCR testing was performed on all BAL samples. For cytomegalovirus (CMV) infection, a cut-off of 10^4^ copies of DNA on BAL was used according to protocols from the lung transplant center. When indicated, RSV infection was treated with oral ribavirin.

Standard bacterial, mycobacterial, and fungal cultures were performed for each sample. A bacterial count of 10^3^ colony-forming units (CFU)/mL is generally considered a positive result for infection, particularly bacterial pneumonia. RealTime PCR was used to identify Mycobacterium tuberculosis complex DNA, and galactomannan detection was performed using a double-sandwich ELISA and considered positive when the results were greater than 0.5 [12]. 

We then analyzed the prevalence of RSV positivity according to the positivity of other tests on the BAL for other viruses, bacteria, and fungi in cases in which these data were retrievable from our electronic record, as previously stated.

### 2.2. Statistics

Sample descriptions are presented as proportions and percentages for categorical variables, with means for continuous variables. The chi-square test and Fisher’s exact test were used to determine differences between categorical variables. A probability (*p*) of less than 0.05 was considered as statistically significant. Statistical analyses were conducted using SPSS software (IBM Corp. Released 2023. IBM SPSS Statistics for Windows, Version 29.0.2.0 Armonk, NY, USA: IBM Corp).

## 3. Results

**Figure 1:**
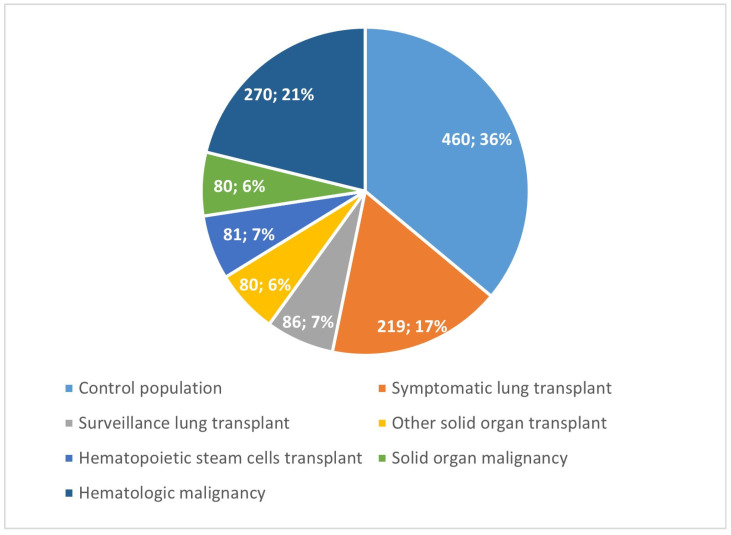
Study population groups.

We collected data from 1319 patients who underwent bronchoscopy with BAL. There were 466 transplant recipients (37% of the population), of which 305 had a lung transplant (further stratified into 219 symptomatic patients and 86 who underwent regular surveillance bronchoscopies), 80 underwent solid organ transplants other than lung, and 81 received hematopoietic stem cell transplants. There were 350 oncologic patients (27% of the population), of which 80 had a diagnosis of solid organ cancer and 270 were affected by hematologic malignancies. The control population consisted of 460 patients (Figure 1).

### 3.1. Incidence of RSV Infection

The overall incidence of RSV infection was 3%, with 40 BALs testing positive (Table 1). Among the control population, there were seven cases of RSV, corresponding to 1.5% incidence. RSV was isolated in 33 immunocompromised patients, with an incidence of 4.1%. Among the lung transplant recipients, the incidence was 3.6% (11 cases), which increased to 5% when considering only symptomatic patients. No cases of RSV positivity were found in lung transplant recipients undergoing surveillance bronchoscopy. Among the SOT (solid organ transplant) recipients, there were two cases of RSV positivity (2.5%). In the hematopoietic stem cell transplant (HCT) recipients, the incidence was 13.6%, with 11 cases. In the oncology patients, the incidence was 2.6%, specifically 1.2% for solid organ cancers (1 case) and 3% for hematologic cancers (8 cases).

### 3.2. Viral Coinfections

Of the 1276 patients for whom tests for viruses other than RSV were available, 441 tested positive (34.6%). No significant correlation was observed between RSV infection and viral co-infections, considered collectively, across the various groups examined. Nevertheless, numerically, viral co-infections were common, with viruses other than RSV isolated in 43.6% of cases; this percentage increased to 50% in the lung transplant recipients and to 54.4% in the stem cell transplant recipients. When evaluating the most frequently isolated viruses, a statistically significantly higher prevalence of RSV was found in the influenza virus infection (strains A and B)-positive patients, with 13.6% of the patients infected with both viruses, compared to influenza-negative patients, for whom the RSV detection rate was 2.7%. This higher prevalence was also present in several additional stratifications, such as in the transplant recipients, particularly in the lung transplant recipients and stem cell transplant recipients. No statistically significant prevalence of CMV co-infection was found in any of the examined populations (Table 2).

### 3.3. Bacterial Coinfections

Of the 1019 patients for whom bacterial cultures were available, 363 tested positive (35.6%). In evaluating the most frequently isolated bacteria, a statistically significant prevalence of coinfections between RSV and *S. pneumoniae* was found. The prevalence of RSV in patients with S pneumoniae was 22.2%, compared to 0.7% of RSV in patients without bacterial infection. This finding was also observed in immunocompromised RSV-positive patients, particularly transplant recipients (33.3% vs. 5.1%) (Table 3). A statistically significant prevalence of *Nocardia* spp. infection was also observed in 66.6% of patients with coinfection of RSV and *Nocardia* spp. vs. 3% of patients with RSV infection alone. Considering the transplant patient population only, the prevalence was 66.6% compared to 4.8% in the control population. All patients with *Nocardia* spp. detection were transplant recipients, including two lung transplant patients and one bone marrow transplant patient. We also found a statistically significant prevalence of co-infections with *M. pneumoniae* and RSV (50%) compared to the prevalence of RSV infection in *M. pneumoniae*-negative patients (3%). However, statistical analysis in subpopulations was not possible due to the small sample size (Table 4). As for the other bacterial isolates analyzed (P. Aeruginosa, S. Aureus, P. Carinii, K. Pneumoniae, A. Xylosoxidans), no statistically significant differences were found.

### 3.4. Fungal Coinfections

Of the 1019 patients for whom tests for the presence of fungal pathogens were available, 43 tested positive (4.2%). The data collected regarding fungal co-infections, specifically *Aspergillus* spp., showed a statistically significant prevalence of RSV infection in positive patients, with a prevalence of 11.6% compared to 2.9% of the fungal infection-negative ones, particularly among transplant recipients (15% vs. 4.8%). Further stratification of the population based on the type of transplant was not possible due to the small sample size (Table 5).

### 3.5. Therapy Efficacy

Of the 40 patients who tested positive for RSV, 5 were treated with ribavirin (12.5%), including 1 lung transplant recipient and 4 stem cell transplant recipients. No statistically significant differences in mortality were observed between RSV-infected patients treated with ribavirin and those receiving only symptomatic treatment (*p* = 0.28). Due to the small sample size, it was not possible to conduct more in-depth analyses by categorizing patients based on the type of transplant.

## 4. Discussion

The main findings of this study, which focuses on BAL results obtained in clinical setting of real-life patient populations, are as follows:

- The incidence of RSV in the immunocompromised patients was higher than in the control population (*p* = 0.01), especially in symptomatic lung transplant patients (*p* < 0.01) and in bone marrow transplant patients (*p* < 0.01)
- Viral coinfections: a high prevalence of influenza virus was found in patients with RSV compared to patients without RSV (*p* < 0.01). These data were also confirmed in transplant recipients (*p* < 0.01)
- Bacterial coinfections: a high prevalence of *S. pneumoniae*, *M. pneumoniae*, and *Nocardia* spp. was found in patients with RSV compared to patients without RSV (*p* < 0.01)
- Mycotic coinfections: a high prevalence of *Aspergillus* spp. was found in patients with RSV compared to patients without RSV (*p* < 0.01), which was also present in the transplant population (*p* = 0.048)

Lung transplant recipients are particularly vulnerable to viral respiratory infections due to their immuno-compromised state. A systematic review found that lung transplant recipients have a significantly increased risk of developing respiratory viral infections compared to the general population [13,14]. In comparison, in the general population, the incidence of RSV infections is much lower and generally limited to seasonal epidemics, with most infections occurring in young children and the elderly [15]. In the general adult population, the incidence of RSV-related hospitalization ranges from 44.2 to 58.9 per 100,000 person-years, with the highest rates in adults aged ≥65 years, ranging from 136.9 to 255.6 per 100,000 person-years [16]. The incidence of RSV infection in lung transplant recipients is between 1800 and 3600 per 100,000 person-years [17]. Evaluating the incidence of RSV infection, in our study, it was noted that, in agreement with the literature, in immunocompromised patients, and more specifically in symptomatic lung transplant patients, there is more frequent isolation of RSV than in the control population. This is a crucial factor in the management of lung transplant recipients. A 2016 study found a lower respiratory trait infection (LRTI) progression rate of 40.1% in lung transplant patients with RSV infection [18]. Moreover, various studies demonstrated that these infections correlate with loss of functionality of the graft and therefore the development of CLAD, a complication with poor prognosis [17,19,20].

In general, no significant correlation was found between RSV infection and viral co-infections, considered as a whole, in the various groups examined. Despite this, numerically, cases of viral co-infection were numerous; in fact, in 43.6% of cases, other viruses were isolated in addition to RSV; a percentage that increased to 50% in the case of lung transplant patients. Evaluating the most frequently isolated viruses, a statistically significant prevalence of influenza virus infection (A and B strains) was found in RSV+ patients compared to RSV– patients: 15.4% of RSV+ patients compared to 3% of the control population. This higher prevalence is also present in further stratifications; for example, in transplant patients (17.4% vs. 1.8%). The relationship between RSV and influenza has been studied extensively, but conflicting data can be found in the literature [21,22,23,24]. In fact, some authors describe a unique interaction between influenza A virus (IAV) and respiratory syncytial virus during coinfection [22]. Using advanced microscopy and imaging techniques, the authors found that human lung cells coinfected with IAV and RSV produce hybrid virus particles (HVPs) that contain surface proteins and ribonucleoproteins from both viruses. These HVPs use the RSV fusion glycoprotein to evade neutralizing anti-IAV antibodies and infect cells lacking IAV receptors. This finding is particularly relevant for lung transplant recipients, who are highly vulnerable to viral infections due to their immunocompromised state. Viral co-infections, such as RSV and influenza, as previously stated, can lead to significant complications, including chronic lung allograft dysfunction (CLAD) and increased mortality [3,7,13,16]. The presence of HVPs could potentially increase the virulence and shedding capacity of viruses, further complicating the clinical management of these infections in lung transplant recipients [20]. Our study also highlights synergic effects of the two viruses, as also stated in a South African study [23]. Another study highlights how, in a population of patients over 65, co-infection between influenza and RSV correlates with a higher risk of severe complications and adverse events, which cannot be explained solely by the age of the patients [24]. On the other hand, other studies support an inhibitory effect between the two viruses, more precisely a protective effect of the influenza virus against RSV infection [25,26,27,28]. This is therefore a topic not yet clarified and of great interest, which requires further evaluation.

Analyzing bacterial co-infections, no significant difference was found in lung transplant patients. By evaluating the most frequently isolated bacteria, a statistically significant prevalence of *S. pneumoniae* co-infections was found in RSV+ patients, where we have a prevalence of 6.1%, compared to RSV– patients, with a prevalence of 0.7%. This trend was also noted when evaluating immunocompromised RSV+ patients, and in particular, transplant patients, comparing them with their respective RSV– cohorts (4.7% vs. 0.5%). It was not possible to conduct further analyses due to the limited number of cases. Moreover, the heterogeneity in microbiological testing should be considered, with a more extensive syndromic panel being used only in critical patients, whereas a limited panel was used as the first choice in uncomplicated transplanted subjects or in controls according to clinical context. The choice of the test also depended on the suspected pathogen; therefore, in case of suspected influenza or RSV, it was sufficient to carry out the Cepheid test without necessarily resorting to more extensive panels. A potential bias in the evaluation of coinfections could be due to the fact that in many cases of GeneXpert Cepheid negativity, FilmArray was sequentially performed. This could represent a potential source of detection bias. On the other hand, a positive result at Ceheid could have precluded subsequent execution of FilmArray. This was considered, taking into account the clinical evaluation. This data, however, agrees with the existing literature. Indeed, a systematic review of 124 articles including pediatric populations identified evidence of direct interactions between RSV and pneumococcus [29]. The evidence implies that RSV and pneumococcus mutually contribute to the incidence and severity of LRTI, and that the elimination or reduction of the prevalence of one of these pathogens attenuates disease associated with the other. Analyzing *Nocardia* spp. infection, a statistically significant prevalence was highlighted in the group of RSV+ patients; this co-infection was found in 6.2% of patients, compared to the RSV– patient cohort, where it was present in only 0.2% of cases. All patients with *Nocardia* spp. were transplant patients, including two lung and one bone marrow recipient. There are currently no data in the literature evaluating this particular type of co-infection; therefore, it would be interesting to explore these data further in the future. A statistically significant prevalence of *M. pneumoniae* co-infection was found in RSV+ patients (6.1%), compared to RSV– patients (0.2%). The studies conducted in this regard are few but provide consistent results; in particular, this study found a significant prevalence of co-infection between RSV and *M. pneumoniae*, especially in the population of patients with COPD [30]. With regard to the other bacterial isolates analyzed (*P. aeruginosa*, *S. aureus*, *P. carinii*, *K. pneumoniae*, *A. xylosoxidans*), no statistically significant differences were found.

Regarding co-infections between *Aspergillus* spp. and RSV, there are few sources in the literature. One study reported that 15% of respiratory viral infection (RVI) episodes in lung transplant recipients are associated with secondary invasive fungal infections (IFI), with Aspergillus being the most common fungal pathogen (80%). Our findings highlight a significant prevalence of fungal co-infections in patients with RSV infections. *Aspergillus* spp. and RSV co-infections in lung transplant recipients are associated with increased morbidity and mortality. Secondary fungal infections have been associated with progression of CLAD and an increased risk of death. In particular, patients with secondary IFIs showed increased disease severity during RVI episodes and an increased risk of CLAD progression or death [31]. Despite the small sample size, the data collected in our center seem to underline a relationship between these two pathogens, with a high rate of co-infections, especially in the population of transplant patients. With the data collected, it was not possible to distinguish the different forms of aspergillosis (e.g., invasive, colonizing, or saprophytic forms). Of note, as discussed above, our study includes data from a real-life clinical setting in which the choice of test was partly linked to clinical judgment. The possibility of bias related to the rate of co-infections must be acknowledged. Our results are exploratory in this sense, and no conclusion on the actual role of immunodeficiency in facilitating co-infections or a role of RSV related to this phenomenon can be drawn. More rigorous studies might be developed in order to address this possibility.

In terms of management, treatment with ribavirin, both oral and inhaled, has been used in multiple studies, although with variable results. One study showed that ribavirin did not prevent lung function decline at 3 months post-infection [19], while another study suggested that ribavirin may be associated with a reduction in the incidence of CLAD [32]. Another study reported that six-month mortality did not differ significantly between patients treated with oral ribavirin and those not treated [33]. Of note, no patients in our sample were treated with IV immunoglobulins. We could not draw any conclusion on the possible role of steroids in a population often requiring it for immunosuppressive reasons. No data on respiratory function or the development of CLAD were available.

The present study has limitations. The data should be interpreted with caution due to the retrospective and monocentric nature of this study. It is possible that the data collected underestimate the true incidence of RSV, since only patients undergoing BAL were considered. We acknowledge that the decision to proceed with BAL may have been influenced by a lower threshold for suspecting infection, which could impact the reported incidence of RSV. While immunocompromised patients require a more aggressive diagnostic approach than the standard, this could be a more relevant bias in a non-immunocompromised population. Furthermore, clinical parameters were not taken into account to assess the severity of the clinical picture. The small sample size also limits the analysis, especially when evaluating subcategories of patients. We were not able to retrieve results for research on other pathogens for all of our patients, limiting the sample for those analyses. There were no defined criteria for the prescription of ribavirin, which was started at the discretion of the physicians, resulting in an uncontrolled analysis of the outcome parameters. Finally, based on the testing availability, diagnostic testing was more comprehensive in transplant patients compared to the control group, possibly leading to an overestimation of co-infection rates in the transplant population. Future research should implement standardized testing protocols (including standardizing the collection of upper and lower respiratory specimens) across immunocompromised and control groups to ensure that all study participants undergo the same comprehensive testing regimen.

## 5. Conclusions

In this study, we observed that immunocompromised patients, such as transplant recipients, face a higher risk of contracting respiratory syncytial virus (RSV) infection compared to the general population when BAL is the method of sample procurement. Further studies with a larger sample size are necessary to confirm these findings and to explore other clinically relevant aspects such as hospitalization rates and the duration of hospital stays related to RSV infections and potential co-infections. Further studies are also needed to deepen our understanding of the correlation between RSV infection and functional alterations or the development of CLAD.

Due to the descriptive and exploratory nature of the findings coming from this single-center study, the true risk based on the data cannot be provided (particularly regarding the role of co-infections and treatment).

This hypothesis-generating study warrants the need for future studies in order to confirm or refute our results. Particularly, the possible role of strategies aimed at preventing RSV infection in such populations might be evaluated in future studies, considering the vulnerability of these patients to RSV infections and the recent advancements in RSV management (e.g., vaccines, immunoglobulins).

## Figures and Tables

**Table 1:** RSV incidence vs. control population. RSV+ is number of positive tests and prevalence in the population.

	Total	RSV+	*p* vs. Control
Control population	460	7 1.5%	
Immunocompromised	802	33 4.1%	0.01
Transplant recipients	466	24 5.1%	<0.01
Lung transplant recipients	305	11 3.6%	ns
Symptomatic lung transplant recipients	219	11 5%	<0.01
Surveillance lung transplant recipients	86	/ /	/
Other solid organs transplant recipients	80	2 2.5%	ns
Hematopoietic cells transplant recipients	81	11 13.6%	<0.01
Oncology	350	9 2.6%	ns
Solid organ malignancy	80	1 1.2%	ns
Hematologic malignancy	270	8 3%	ns

**Table 2:** Viral coinfections: for each group of subjects, the total number of tests, the number of tests in subjects who tested positive for RSV, and the percentage of the group population.

	Test for Other Viruses			Test for CMV			Test for Influenza		
	Neg	POS	*p*	Neg	Pos	*p*	Neg	Pos	*p*
Total population, n	835	441	ns	1146	130	ns	1232	44	0.001
Positive test for RSV, n (%)	22 (2.6)	17 (3.8)		33 (2.9)	6 (4.6)		33 (2.7)	6 (13.6)	
Immunocompromised, n	512	276	ns	690	98	ns	759	29	0.001
Positive test for RSV, n (%)	18 (3.6)	14 (5.1)		27 (3.9)	5 (5.1)		27 (3.5)	5 (17.2)	
Transplant, n	277	179	ns	377	79	ns	444	12	0.001
Positive test for RSV, n (%)	12 (4.5)	11 (6.5)		18 (4.8)	5 (6.3)		19 (4.3)	4 (33.3)	
Lung transplant, n	183	116	ns	239	60	ns	/	/	/
Positive test for RSV, n (%)	5 (2.7)	5 (4.3)		7 (2.9)	3 (5)				
Symptomatic lung tx, n	138	76	ns	177	37	ns	/	/	/
Positive test for RSV, n (%)	5 (3.6)	5 (6.6)		7 (3.9)	3 (8.1)				
Surveillance lung tx, n	/	/	/	/	/	/	/	/	/
Positive test for RSV, n (%)									
Other solid organs tx, n	/	/	/	/	/	/	/	/	/
Positive test for RSV, n (%)									
Hematopoietic cells tx, n	47	32	ns	69	10	ns	73	6	0.001
Positive test for RSV, n (%)	5 (10.6)	6 (18.7)		9 (13.0)	2 (20.0)		7 (9.6)	4 (66.6)	
Oncologic, n	238	107	ns	/	/	/	327	18	ns
Positive test for RSV, n (%)	6 (2.5)	3 (2.9)					8 (4.4)	1 (5.5)	
Solid organ malign, n	/	/	/	/	/	/	/	/	/
Positive test for RSV, n (%)									
Hematologic malign, n	189	78	ns	/	/	/	251	16	ns
Positive test for RSV, n (%)	5 (2.6)	3 (3.8)					7 (2.8)	1 (6.2)	

**Table 3:** Bacterial coinfections: for each group of subjects, the total number of tests, the number of tests in subjects who tested positive for RSV, and the percentage of the group population.

	Test for Bacteria			Test for *S. pneumoniae*		
	Neg	Pos	*p*	Neg	Pos	*p*
Total population	656	363	ns	1010	9	0.001
Positive test for RSV, n (%)	16 (2.4)	17 (4.7)		31 (3.1)	2 (22.2)	
Immunocompromised	444	231	ns	671	4	0.041
Positive test for RSV, n (%)	15 (3.4)	14 (6.1)		28 (4.2)	1 (25)	
Transplant	239	156	ns	392	3	0.030
Positive test for RSV, n (%)	12 (5.0)	9 (5.8)		20 (5.1)	1 (33.3)	
Lung transplant	152	110	ns	/	/	/
Positive test for RSV, n (%)	5 (3.3)	4 (3.6)				
Symptomatic lung tx	95	83	ns	/	/	/
Positive test for RSV, n (%)	5 (5.3)	4 (4.8)				
Surveillance lung tx	/	/	/	/	/	/
Positive test for RSV, n (%)						
Other solid organs tx	/	/	/	/	/	/
Positive test for RSV, n (%)						
Hematopoietic cells tx	55	19	ns	/	/	/
Positive test for RSV, n (%)	6 (10.9)	5 (26.3)				
Oncologic	214	77	0.02	/	/	/
Positive test for RSV, n (%)	3 (1.4)	5 (6.5)				
Solid organ malign.	/	/	/	/	/	/
Positive test for RSV, n (%)						
Hematologic malign.	179	54	0.03	/	/	/
Positive test for RSV, n (%)	3 (1.7)	4 (7.4)				

**Table 4:** Nocardia and *M. pneumoniae* coinfections: for each group of subjects, the total number of tests, the number of tests in subjects who tested positive for RSV, and the percentage of the group population.

	Test for Nocardia			Test for *M. pneumoniae*		
	Neg	Pos	*p*	Neg	Pos	*p*
Total population	1016	3	0.003	1015	4	0.006
Positive test for RSV, n (%)	31 (3.0)	2 (66.7)		31 (3.0)	2 (50.0)	
Immunocompromised	672	3	0.005	/	/	/
Positive test for RSV, n (%)	27 (4.0)	2 (66.6)				
Transplant	392	3	0.01	/	/	/
Positive test for RSV, n (%)	19 (4.8)	2 (66.6)				
Lung transplant	260	2	ns	/	/	/
Positive test for RSV, n (%)	8 (3.1)	1 (50.0)				
Symptomatic lung tx	/	/	/	/	/	/
Positive test for RSV, n (%)						
Surveillance lung tx	/	/	/	/	/	/
Positive test for RSV, n (%)						
Other solid organ tx	/	/	/	/	/	/
Positive test for RSV, n (%)						
Hematopoietic cells tx	/	/	/	/	/	/
Positive test for RSV, n (%)						
Oncologic	/	/	/	/	/	/
Positive test for RSV, n (%)						
Solid organs malign.	/	/	/	/	/	/
Positive test for RSV, n (%)						
Hematologic malign.	/	/	/	/	/	/
Positive test for RSV, n (%)						

**Table 5:** Aspergillus coinfection: for each group of subjects, the total number of tests, the number of tests in subjects who tested positive for RSV, and the percentage over the group population.

	Test for Aspergillus		
	Neg	Pos	*p*
Total population	976	43	0.001
Positive test for RSV, n (%)	28 (2.9)	5 (11.6)	
Immunocompromised	639	36	0.038
Positive test for RSV, n (%)	25 (3.9)	4 (11.1)	
Transplant	375	20	0.048
Positive test for RSV, n (%)	18 (4.8)	3 (15.0)	
Lung transplant	/	/	/
Positive test for RSV, n (%)			
Symptomatic lung tx	/	/	/
Positive test for RSV, n (%)			
Surveillance lung tx	/	/	/
Positive test for RSV, n (%)			
Other solid organ tx	/	/	/
Positive test for RSV, n (%)			
Hematopoietic cells tx	68	6	ns
Positive test for RSV, n (%)	9 (13.2)	2 (33.3)	
Oncologic	274	17	ns
Positive test for RSV, n (%)	7 (2.5)	1 (5.9)	
Solid organs malign.	/	/	/
Positive test for RSV, n (%)			
Hematologic malign.	219	14	ns
Positive test for RSV, n (%)	6 (2.7)	1 (7.1)	

## Data Availability

The data are available upon request due to restrictions (privacy).

## Abbreviations

The following abbreviations are used in this manuscript:

BAL	Bronchoalveolar lavage
RSV	Respiratory syncytial virus
CLAD	Chronic lung allograft dysfunction
SOT	Solid organ transplant
HCT	Hemopoietic cell transplant
IFI	Invasive fungal infection
RVI	Respiratory virus infection
IAV	Influenza A virus
HVP	Hybrid virus particle
LRTI	Lower respiratory tract infection
